# MiR-194-5p enhances the sensitivity of nonsmall-cell lung cancer to doxorubicin through targeted inhibition of hypoxia-inducible factor-1

**DOI:** 10.1186/s12957-021-02278-3

**Published:** 2021-06-14

**Authors:** Mengning Xia, Lili Sheng, Wei Qu, Xue Xue, Hucheng Chen, Guoyan Zheng, Weigang Chen

**Affiliations:** 1 Department of Ultrasound, Children’s Hospital of Nanjing Medical University, Nanjing, 210006 People’s Republic of China; 2 Department of Blood Transfusion, Nanjing Benq Medical Center, Nanjing, 210021 People’s Republic of China; 3 Department of Nuclear Medicine, Nanjing First Hospital, Nanjing Medical University, Nanjing, 210006 People’s Republic of China; 4 Laboratory Medical Center, The Second Affiliated Hospital of Nanjing Medical University, Nanjing, Jiangsu Province 210000 People’s Republic of China

**Keywords:** Doxorubicin resistance, HIF-1, miR-194-5p, NSCLC

## Abstract

**Background:**

Despite chemotherapy being a common treatment, an increase in chemoresistance over time is unavoidable. We therefore investigated the role of miR-194-5p in regulating chordoma cell behavior and examined the downstream effectors of miR-194-5p.

**Methods:**

In this study, NSCLC cell lines A549 and H460 were cultured under hypoxic conditions for 1 week to induce drug resistance to doxorubicin (DOX). The connection between miR-194-5p and HIF-1 was revealed by reverse transcription and real-time polymerase chain reaction (RT-qPCR), western blot, and dual-luciferase assays. We used TUNEL staining and the CCK-8 test to assess the sensitivity of NSCLC cells to DOX.

**Results:**

We found that hypoxia-induced NSCLC cells enhanced resistance to DOX. MiR-194-5p was substantially reduced, and HIF-1 was increased in hypoxia-induced drug-resistant NSCLC cells. Moreover, miR-194-5p successfully induced NSCLC cell apoptosis by directly inhibiting HIF-1, thereby enhancing DOX sensitivity.

**Conclusions:**

MiR-194-5p enhanced the sensitivity of NSCLC cells to DOX by directly inhibiting HIF-1. This work provides insights into underlying treatments for drug-resistant NSCLC.

## Background

Lung cancer is a malignant disease that has the maximum rate of fatality of all cancers globally [1]. As revealed by GLOBOCAN 2018, among the projected 18.1 million new incidences of cancer in 2018, lung cancer patients accounted for 11.6%; lung cancer mortality accounted for 18.4% of the total cancer deaths, ranking first [2]. Similarly, in the cancer statistics from 2014, there were approximately 781,000 new lung cancer cases in China, making it the most common cancer domestically [3]. Although various targeted drugs have shown significant therapeutic effects on nonsmall-cell lung cancer (NSCLC) patients in clinical practice, most patients still inevitably developed acquired resistance to these treatments [4, 5]. Doxorubicin (DOX) is a chemotherapeutic drug that has been widely used to treat a variety of malignant tumors; however, its therapeutic effects have been weakened due to the development of drug resistance [6]. Therefore, investigating the molecular mechanisms of DOX-induced tumor cell apoptosis can be conducive to the targeted development of drugs with high sensitivity to NSCLC cells as part of combined strategies. Such research may shed new light on the treatment of this malady.

Studies have shown that in solid tumors such as NSCLC and liver cancer, the tumor microenvironment is in a state of hypoxia for a long time [7]. In some cases, hypoxia can result in drug resistance to radiotherapy and chemotherapy and promote cancer cell metastasis, featuring the lack of HIF-1, which seems to be an essential link [8]. Studies have shown that hypoxia-induced drug resistance is mediated by the activation of HIF-1*α*; this results in P-glycoprotein (P-gp) protein overexpression, which is a hallmark of hypoxia-induced drug resistance [9]. In addition, the inhibition of tumor cell apoptosis is another important mechanism behind this process [10]. It has also been demonstrated that p53 participates in hypoxia-induced chemoresistance of cancer cells by regulating HIF-1 and P-gp levels [11, 12].

MiRNAs, as small, highly conserved endogenous noncoding RNAs, are known to cut and degrade target messenger RNAs (mRNAs), inhibiting their translational ability to regulate gene expression [13]. MiRNAs commonly affect the pathogenesis of cancers as oncogenes or suppressers and play a vital role [14]. As demonstrated in several studies, the various miRNAs that are of significance to the progression of cancers are poorly regulated [15, 16]. Certain miRNAs are considered potential biomarkers to diagnose and treat NSCLC [17]. Studies have reported that lncRNA TUG1 regulates CCND2 by inhibiting miR-194-5p, thereby promoting the growth and drug resistance of bladder cancer cells [18]. Moreover, miR-194-5p can inhibit the expression level of FOXA1 in NSCLC cells, thereby promoting the sensitivity of NSCLC cells to cisplatin [19]. However, the specific mechanism of miR-194-5p in NSCLC multidrug resistance remains to be elucidated. Therefore, this study further explored the role and molecular mechanism of miR-194-5p in hypoxia-induced DOX resistance in NSCLC.

This paper revealed the considerable downregulation of miR-194-5p expression in hypoxic-induced DOX-resistant NSCLC cells. We further proved that HIF-1 can serve as an immediate-acting subject aimed at miR-194-5p using a double-luciferase test. As demonstrated, miR-194-5p directly inhibited HIF-1, subsequently inhibiting P-gp expression to improve the chemical responsiveness of NSCLC cells to DOX. Therefore, NSCLC cell apoptosis was triggered. In summary, the specific molecular mechanism of miR-194-5p in promoting the chemical responsiveness of NSCLC cells to DOX was elaborated in this paper, which could assist medical professionals in deciding how to treat drug-resistant NSCLC.

## Methods

### Cell growth and processing

Human NSCLC cell lines H460 and A549 were supplied by the Chinese Academy of Sciences (Shanghai, China). DMEM (Life Technologies, CA, USA) containing 10% FBS, 1% penicillin, and 1% streptomycin (Invitrogen, Carlsbad, CA, USA) was used for cell culture in a constant temperature incubator of 5% CO_2_ at 37°C.

### Cell transfection

MiR-NC, a miR-194-5p mimic, was provided by Ambion (Austin, TX, USA). MiR-194-5p mimic, inhibitory substance, or negative regulation were weakened at room temperature (RT) in Opti-MEM medium (Life Technologies, CA, USA) for 15 min, and miR-194-5p mimic or inhibitor was transfected into human NSCLC cells, which were then cultured for 48 h. miR-194-5p expression was determined by qRT-PCR. The process of nucleic acid transfer was carried out using Lipofectamine RNAiMAX reagent (Invitrogen, CA, USA) according to the guidance from the supplier. The expression of HIF-1 was detected through RT-qPCR and western blot analysis.

### Western blot

All cell extracts were obtained by lysing cells in ice-cold lysis buffer. The undissolved matter was removed by high-speed centrifugation, and the protein percentage of the supernatant was measured by a BCA protein test kit. Each group of samples was loaded on a 10% SDS-PAGE gel, and the bands were transferred to a PVDF membrane. After being blocked with the sealing solution, the membranes were immunolabeled overnight with the following primary antibodies: HIF-1 (1:1000, Cell Signaling Technology, USA), p53 (1:1000, Cell Signaling Technology, USA), P-gp (1:1000, Cell Signaling Technology, USA), Bax (1:1000, Abcam, USA), F-Caspase3 (1:1000, Cell Signaling Technology, USA), C-Caspase3 (1:1000, Cell Signaling Technology, USA), F-Casase9 (1:1000, Cell Signaling Technology, USA), C-Caspase9 (1:1000, Cell Signaling Technology, USA), F-PARP (1:1000, Abcam, USA), C-PARP (1:1000, Abcam, USA), and β-actin antibodies (1:1000, Santa Cruz, USA). After washing, the protein bands on the PVDF membrane were incubated with suitable auxiliary antibodies (1:2000, ICN Pharmaceuticals), while an ECL kit was used for color development. The bands were analyzed after exposure to a ChemiDoc XRS+ gel imager (Bio-Rad, USA), and the protein content was expressed as the relative value of the corresponding internal reference band.

### TUNEL staining

To detect apoptosis, the terminal colorimetric TUNEL in situ apoptosis examination kit was applied (Promega, WI, USA), which was tested in line with the requirements of the supplier. The cells were fixed in 4% (w/v) paraformaldehyde for 30 min before washing in PBS for 5 min. They were incubated at 20 μg/mL protease K for 10 min before washing in PBS and were incubated with 3% hydrogen peroxide for 5 min and washed with PBS to inhibit endogenous peroxidase activity. The cells were immersed in equilibration solution for 5 min before incubation with terminal deoxynucleotidyl transferase (TdT) enzyme at 37 °C for 60 min. TdT was used as a negative control before the reaction was completed. After being treated with TdT, the cells were administered 2 × SCC for 15 min and washed with PBS 3 times for 5 min each time. Streptavidin peroxidase was used for 45 min before washing the cells with PBS. The visualization of apoptotic cells was conducted with diaminobenzidine (DAB) (00-2020), and counterstaining was performed for Meyer’s hematoxylin staining (72804E; Microm, CEO Kong Hanning, Germany). The cells were washed in distilled water and installed in aqueous media. An Olympus BX40 light microscope was used for observation. To analyze the apoptosis index (AI), we counted TUNEL-positive cells in 10 regions and used the formula below to obtain the proportion of stained apoptotic cells: AI = (the number of apoptotic cells/total cells) × 100.

### Validation of cell viability

Cell viability was assessed by a CCK-8 kit (Promega, USA) in line with the supplier’s guidance. In simple terms, human OS cells after transfection were implanted into 96-well plates, and attachment was permitted throughout the night. Freshly derived mimics, inhibitors or siRNA were combined with the wells based on the plan and subjected to incubation for an extra72 h. The CCK-8 solution was combined with a 96-well plate, and cells were incubated for an additional 2 h at 37°C. Finally, the absorbance at 450 nm was assessed by a microplate reader.

### RNA sample collection and qRT-PCR

Total RNA was isolated from controls or transfected cells by the RNeasy kit (Qiagen, USA), and miRNA supplementary DNA (cDNA) was transcribed from total RNA using the cDNA reverse transcription kit (Takara Bio, Korea). Real-time PCR was performed by IQ SYBR Green Supermix (1708886, Bio-Rad, USA) in line with the supplier’s guidance. The primers below were applied: miR-194-5p forward 5′-CTAGTACCTAGAGGAACCTTTGAAGACTGTTACAGCTCAGCA-3′, reverse 5′-AGCTTGCTGAGCTGTAACAGTCTTCAAAGGTTCCTCTAGGTA-3′; *GAPDH* forward 5′-CCCACTCCTCCACCTTTGAC-3′, reverse 5′-CCACCACCCTGTTGCTGTAG-3′. *GAPDH* was used as a loading control, and the comparative gene manifestation was determined by the ΔΔCT method. The qPCR assays were conducted three times.

### Luciferase reporter assay

A 3′UTR fraction from the HIF-1 gene was amplified from genomic DNA by PCR and contained a predicted binding site for miR-194-5p. The fraction after amplification was replicated into a UTR downstream of the luciferase gene in a pMIR-reporter luciferase vector (Ambion, USA). Suitable mutation constructs were applied to exert control. NSCLC cells were cotransfected with the test luciferase reporter plasmid and the Renilla luciferase plasmid. Subsequently, the cells were obtained, the dual luciferase activity was evaluated by the Dual-Glo luciferase test regimen, and Renilla was used to exercise intrinsic regulation.

### Data analysis

GraphPad Prism software (version 8.0 GraphPad software) was applied to conduct data analysis. The data are shown as the mean ± standard deviation (SD). Disparities in all indices among batches were assessed using unpaired two-tailed Student’s *t*-tests or two-way ANOVA followed by post hoc *t*-tests (Bonferroni’s or Dunnett’s test). A *P* value of < 0.05 was treated as statistically significant.

## Results

### Downregulation of miR-194-5p and upregulation of HIF-1 in hypoxia-induced NSCLC cells

**Fig. 1:**
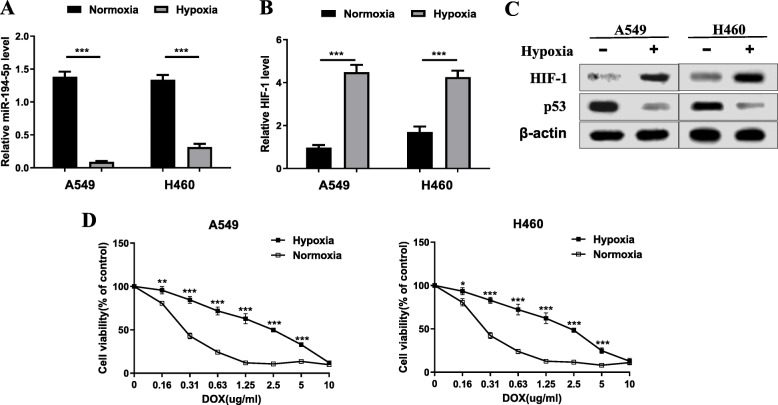
Downregulation of miR-194-5p and upregulation of HIF-1 in hypoxia-induced NSCLC cells. **a**, **b** RT-qPCR was used to detect the expression levels of miR-194-5p and HIF-1 in hypoxia-induced NSCLC cell lines A549 and H460. **c** The expression of HIF-1 in hypoxia-induced NSCLC cell lines A549 and H460 was detected by western blot. **d** The CCK-8 method was performed to detect the viability of hypoxia-induced NSCLC cells and control NSCLC cells under different concentrations of DOX treatment. Data are presented as the mean ± SD. *P <0.05; **P <0.01; ***P <0.001

Allowing for the dysregulation of miRNAs under hypoxic conditions in cancers, we detected the detailed change in miR-194-5p levels in hypoxia-induced disorders in NSCLC. First, we examined hypoxia-induced NSCLC cell lines A549 and H460 using RT-qPCR analysis. The miR-194-5p transcription level showed a sharp decline in hypoxia-induced A549 and H460 cells, while the transcription level of HIF-1 increased (Fig. 1a, b). In addition, western blot analysis demonstrated that the degree of HIF-1 manifestation was noticeably upregulated in hypoxia-induced A549 and H460 cells, while the degree of p53 manifestation experienced considerable downregulation (Fig. 1c). Second, the viability of hypoxia-induced A549 and H460 cells and negative controls treated with DOX at different concentrations was detected by CCK-8 assay, and it was found that the survival ability of hypoxia-induced NSCLC cells was enhanced (Fig. 1d).

### The overexpression of miR-194-5p ameliorated DOX resistance in NSCLC cells induced by hypoxia

**Fig. 2:**
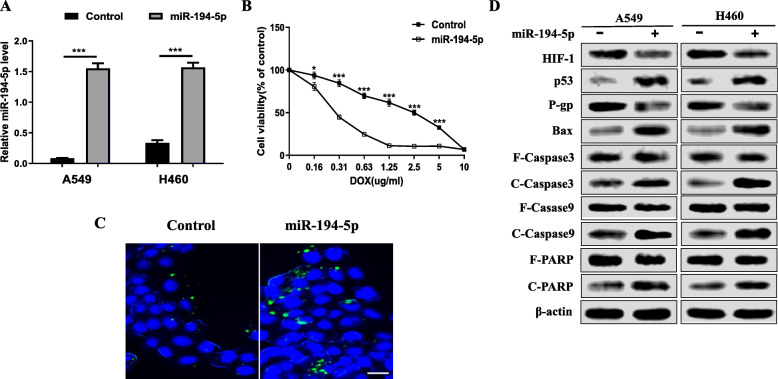
The overexpression of miR-194-5p ameliorated DOX resistance in NSCLC cells induced by hypoxia. **a** The transcription of miR-194-5p in A549 miR-194-5p-overexpressing NSCLC cells was detected by RT-qPCR. **b** The CCK-8 method was used to detect the viability of miR-194-5p-overexpressing NSCLC cells and control NSCLC cells induced by hypoxia under different concentrations of DOX treatments. **c** TUNEL staining was used to detect the apoptosis rate of miR-194-5p-overexpressing NSCLC cells and control NSCLC cells (bar=25 μm). **d** The expression levels of HIF-1, BAX, Caspase-9, Caspase-3, PARP, and p-gp in miR-194-5p-overexpressing NSCLC cells and control NSCLC cells induced by hypoxia were detected by western blot. Data are presented as the mean ± SD. *P <0.05; ***P < 0.001

For an in-depth investigation into the specific difference made by miR-194-5p in hypoxic NSCLC cells, miR-194-5p in A549 cells was overexpressed by plasmid transfection. The transcription level of miR-194-5p increased in hypoxic A549 cells (Fig. 2a). The viability of hypoxic NSCLC cells and negative controls treated with DOX at different concentrations was measured by the CCK-8 assay. miR-194-5p overexpression weakened the viability of hypoxic A549 cells treated with DOX (Fig. 2b). In addition, TUNEL staining showed that the overexpression of miR-194-5p increased the apoptosis rate of hypoxic A549 cells treated with DOX (Fig. 2c). In addition, the protein levels in the typical mitochondrial apoptosis pathway were detected. Western blot analysis revealed that miR-194-5p overexpression reduced HIF-1 and P-gp levels in hypoxic A549 cells. The degrees of BAX, cleaved caspase-9, cleaved caspase-3, and cleaved PARP were increased, indicating the activation of p53 and mitochondrial apoptotic pathways (Fig. 2d).

### HIF-1 as an immediate target of miR-194-5p

**Fig. 3:**
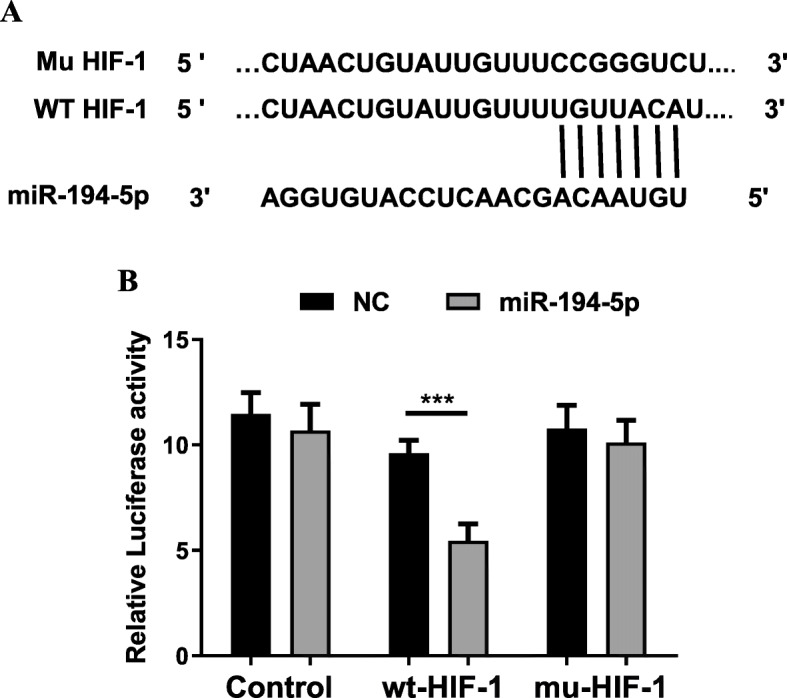
HIF-1 as an immediate target of miR-194-5p*.* **a** The target binding sequence of HIF-1 and miR-194-5p was predicted from the TargetScan database (http://www.targetscan.org). **b** A dual luciferase reporter assay was conducted to evaluate the luciferase activities of WT HIF-1 or MUT HIF-1 and miR-194-5p or *miR-NC* cotransfected A549 cells. Data are presented as the mean ± SD. ***P < 0.001

To explore how miR-194-5p relates to HIF-1, we performed miRNA predictions using TargetScan. HIF-1 was discovered to be an immediate target of the gene. The predicted binding sequence is listed in Fig. 3a. Furthermore, we performed a luciferase reporter assay to establish whether miR-194-5p relates to HIF-1. By amplifying the 3′UTR of human HIF-1 and cloning the resulting fragment into the pmirGLO vector, the target region predicted by miR-194-5p in the 3′UTR of HIF-1 was mutated. The HIF-1 3′UTR plasmid and miR-194-5p mimic were cotransfected into A549 cells. As a result, relative to the control batch, the luciferase activity experienced a significant reduction in the cotransfected A549 cells, indicating that the HIF-1 level was affected by the mRNA level. The negative regulation of miR-194-5p and the cotransfected Mu-HIF-1 3′UTR plasmid and miR-194-5p mimics into A549 cells restored luciferase activity. All this indicates that HIF-1 was identified as an immediate target of miR-194-5p (Fig. 3b).

### Mandatory overexpression of HIF-1 reversed the chemosensitization effect of miR-194-5p upregulation

**Fig. 4:**
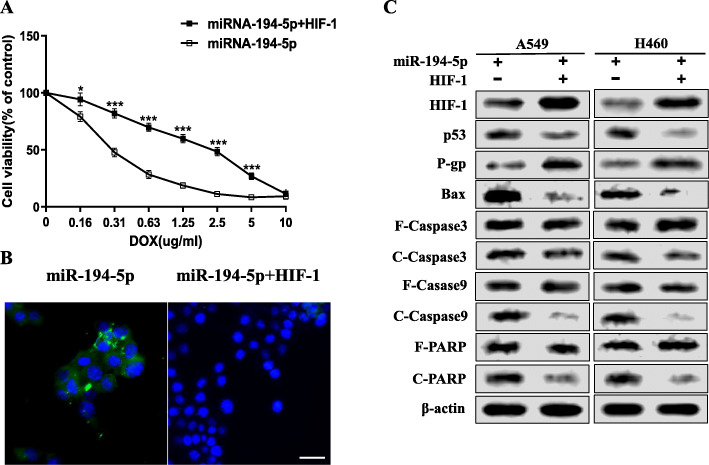
Mandatory overexpression of HIF-1 reversed the chemosensitization effect of miR-194-5p upregulation. **a** A CCK-8 cell viability test was performed to detect the survival ability of HIF-1-overexpressing A549 cells under different concentrations of DOX treatments. **b** TUNEL staining was used to detect the apoptosis rate of HIF-1-overexpressing A549 cells and control NSCLC cells (Bar=25 μm). **c** The expression levels of HIF-1, BAX, Caspase-9, Caspase-3, PARP, and p-gp in HIF-1-overexpressing NSCLC cells and miR-194-5p-overexpressing NSCLC cells induced by hypoxia were detected by western blot. Data are presented as the mean ± SD. *P <0.05; ***P < 0.001

To explore the role played by HIF-1 and miR-194-5p in hypoxia-induced A549 cells, the HIF-1 plasmid was transfected into miR-194-5p-overexpressing A549 cells to force the overexpression of HIF-1. A CCK-8 cell viability test was performed, and we found that the survival ability of HIF-1-overexpressing A549 cells was enhanced after DOX administration (Fig. 4a). Similarly, TUNEL staining analysis showed that hypoxia-induced HIF-1-overexpressing A549 cells had a significant reduction after DOX administration (Fig. 4b). Western blot analysis demonstrated that the expression of HIF-1 and P-gp was upregulated in HIF-1-overexpressing A549 cells, while that of BAX, cleaved caspase-9, cleaved caspase-3, and cleaved PARP was suppressed (Fig. 4c). These results suggest that the activation of p53 and mitochondrial apoptotic pathways was repressed, completely reversing the effect caused by miR-194-5p overexpression.

## Discussion

Nonsmall-cell lung cancer (NSCLC) is a common malignant tumor characterized by high morbidity and high mortality. Because of the multiple gene mutation types and the heterogeneity of its associated tumors, NSCLC easily develops drug resistance through genetic diversity, which poses challenges to its treatment [20].

Chemotherapy is an important clinical procedure for NSCLC treatment. DOX has been widely applied clinically as a broad-spectrum tumor chemotherapy drug. It exerts antitumor effects by inducing tumor cell apoptosis, autophagy, and necrosis [21]. However, due to the heterogeneity of NSCLC cells, multiple mutations in the NSCLC genome can be found in patients receiving chemotherapeutic medications, and the resulting drug resistance further thwarts the therapeutic effects, making long-term disease control unattainable. However, drug resistance can seriously limit the clinical therapeutic efficacy of DOX treatment in various cancers, including NSCLC [22]. Therefore, exploring the molecular mechanisms that can kill DOX-resistant NSCLC cells is of great significance for patients with advanced NSCLC. Our present study revealed that miR-199a-5p was essential for D resistance in NSCLC cells by regulating the expression of HIF-1α.

In recent years, progress has been made in the early diagnosis and treatment of NSCLC, but the desired achievements have not been attained [23]. With the rapid development of bioinformatics, microRNAs that are clearly associated with the progression of NSCLC are easier to identify [24]. miR-194 has shown potential tumor suppressor effects in many cancers. For example, miR-194 inhibits the migration, invasion, and epithelial-mesenchymal transition (EMT) of gastric cancer cells by downregulating FoxM1 [25]. miR-194 directly inhibits the expression of CDH2 to reduce the proliferation and migration of osteosarcoma cells and promote cell apoptosis [26]. For patients with advanced colorectal adenoma after polypectomy, miR-194 can be used as a promising biomarker to judge the prognosis of adenoma recurrence [27]. Recently, many studies have reported the important role of miR-194-5p in chemotherapy resistance. Its effect on tumor chemotherapy resistance varies among different cancers and different drugs. For example, miR-194-5p can induce cisplatin resistance in ovarian cancer by inhibiting the expression of SLC40A1 [28]. There are also other reports indicating that miR-194-5p induces p21 upregulation and G1 phase arrest in drug-resistant cells by downregulating MDM2, thereby resensitizing drug-resistant ovarian cancer cells to paclitaxel [29]. Another study showed that miR-194-5p can inhibit the expression level of FOXA1 in NSCLC cells, thereby promoting the sensitivity of NSCLC cells to cisplatin [19]. However, its particular role in the underlying mechanism in NSCLC remains unclear.

In this study, A549 and H460 NSCLC cell lines were cultured for 7 days under hypoxic conditions to construct a DOX-resistant NSCLC cell model. RT-qPCR analyses demonstrated that the transcription degree of miR-194-5p in hypoxia-induced NSCLC cells was downregulated, while miR-194-5p overexpression repressed HIF-1 and P-gp levels in hypoxic A549 cells. Upregulation of BAX, cleaved caspase-9, cleaved caspase-3, and cleaved PARP activated p53 and mitochondrial apoptotic pathways, hence promoting hypoxia-induced NSCLC cell apoptosis after DOX treatment. The luciferase reporter assay further confirmed that miR-194-5p enhanced the DOX sensitivity of NSCLC cells by directly inhibiting HIF-1. Forced overexpression of HIF-1 repressed the enhanced DOX sensitivity resulting from miR-194-5p overexpression. As the mechanism has been clarified above, we confirmed that miR-194-5p augments the responsiveness of NSCLC cells to DOX through HIF-1 downregulation.

## Conclusions

In summary, miR-194-5p can directly inhibit HIF-1 and regulate the expression of a series of downstream proteins including BAX, cleaved caspase-9, cleaved caspase-3, cleaved PARP, and P-gp, thus enhancing the therapeutic effect of DOX on NSCLC cells and promoting NSCLC cell apoptosis. The outcomes show that miR-194-5p may be a potential target for overcoming NSCLC multidrug resistance and is expected to become a new therapeutic target for mitigating drug resistance in NSCLC.

## Data Availability

The datasets used and/or analyzed during the current study are available from the corresponding author on reasonable request.

## References

[1] Stockwell BR, Friedmann Angeli JP, Bayir H, Bush AI, Conrad M, Dixon SJ, Fulda S, Gascón S, Hatzios SK, Kagan VE, Noel K, Jiang X, Linkermann A, Murphy ME, Overholtzer M, Oyagi A, Pagnussat GC, Park J, Ran Q, Rosenfeld CS, Salnikow K, Tang D, Torti FM, Torti SV, Toyokuni S, Woerpel KA, Zhang DD (2017). Ferroptosis: a regulated cell death nexus linking metabolism, redox biology, and disease. Cell..

[2] Bray F, Ferlay J, Soerjomataram I, Siegel RL, Torre LA, Jemal A (2018). Global cancer statistics 2018: GLOBOCAN estimates of incidence and mortality worldwide for 36 cancers in 185 countries. CA Cancer J Clin.

[3] Chen W, Sun K, Zheng R, Zeng H, Zhang S, Xia C, Yang Z, Li H, Zou X, He J, National Office for Cancer Prevention and Control, National Cancer Center/Cancer Hospital, Chinese Academy of Medical Sciences and Peking Union Medical College, Beijing 100021, China (2018). Cancer incidence and mortality in China, 2014. Chin J Cancer Res.

[4] Lynch TJ, Bell DW, Sordella R, Gurubhagavatula S, Okimoto RA, Brannigan BW, Harris PL, Haserlat SM, Supko JG, Haluska FG, Louis DN, Christiani DC, Settleman J, Haber DA (2004). Activating mutations in the epidermal growth factor receptor underlying responsiveness of non-small-cell lung cancer to gefitinib. N Engl J Med.

[5] Tan CS, Gilligan D, Pacey S (2015). Treatment approaches for EGFR-inhibitor-resistant patients with non-small-cell lung cancer. Lancet Oncol.

[6] Wang J, Feng C, He Y, Ding W, Sheng J, Arshad M, Zhang X, Li P (2015). Phosphorylation of apoptosis repressor with caspase recruitment domain by protein kinase CK2 contributes to chemotherapy resistance by inhibiting doxorubicin induced apoptosis. Oncotarget..

[7] Tao L, Shu-Ling W, Jing-Bo H, Ying Z, Rong H, Xiang-Qun L, Wen-Jie C, Lin-Fu Z (2020). MiR-451a attenuates doxorubicin resistance in lung cancer via suppressing epithelialmesenchymal transition (EMT) through targeting c-Myc. Biomed Pharmacother.

[8] Balamurugan K (2016). HIF-1 at the crossroads of hypoxia, inflammation, and cancer. Int J Cancer.

[9] Kim HG, Hien TT, Han EH, Hwang YP, Choi JH, Kang KW, Kwon KI, Kim BH, Kim SK, Song GY, Jeong TC, Jeong HG (2011). Metformin inhibits P-glycoprotein expression via the NF-kappaB pathway and CRE transcriptional activity through AMPK activation. Br J Pharmacol.

[10] Xu RH, Pelicano H, Zhou Y, Carew JS, Feng L, Bhalla KN, Keating MJ, Huang P (2005). Inhibition of glycolysis in cancer cells: a novel strategy to overcome drug resistance associated with mitochondrial respiratory defect and hypoxia. Cancer Res.

[11] Erler JT, Cawthorne CJ, Williams KJ, Koritzinsky M, Wouters BG, Wilson C, Miller C, Demonacos C, Stratford IJ, Dive C (2004). Hypoxia-mediated down-regulation of Bid and Bax in tumors occurs via hypoxia-inducible factor 1-dependent and -independent mechanisms and contributes to drug resistance. Mol Cell Biol.

[12] Kim HS, Wannatung T, Lee S, Yang WK, Chung SH, Lim JS, Choe W, Kang I, Kim SS, Ha J (2012). Quercetin enhances hypoxia-mediated apoptosis via direct inhibition of AMPK activity in HCT116 colon cancer. Apoptosis..

[13] Macfarlane LA, Murphy PR (2010). MicroRNA: biogenesis, function and role in cancer. Curr Genom.

[14] Lee YS, Dutta A (2009). MicroRNAs in cancer. Annu Rev Pathol.

[15] Nijhuis A, Thompson H, Adam J, Parker A, Gammon L, Lewis A, Bundy JG, Soga T, Jalaly A, Propper D, Jeffery R, Suraweera N, McDonald S, Thaha MA, Feakins R, Lowe R, Bishop CL, Silver A (2017). Remodelling of microRNAs in colorectal cancer by hypoxia alters metabolism profiles and 5-fluorouracil resistance. Hum Mol Genet.

[16] Kim SW (2017). The role of microRNAs in colorectal cancer. Korean J Gastroenterol.

[17] Muller S, Janke F, Dietz S, Sultmann H (2020). Circulating microRNAs as potential biomarkers for lung cancer. Recent Results Cancer Res.

[18] Yu G, Zhou H, Yao W, Meng L, Lang B (2019). lncRNA TUG1 promotes cisplatin resistance by regulating CCND2 via epigenetically silencing miR-194-5p in bladder cancer. Mol Ther Nucleic Acids.

[19] Zhu X, Li D, Yu F, Jia C, Xie J, Ma Y, Fan S, Cai H, Luo Q, Lv Z, Fan L (2016). miR-194 inhibits the proliferation, invasion, migration, and enhances the chemosensitivity of non-small cell lung cancer cells by targeting forkhead box A1 protein. Oncotarget..

[20] Wald P, Liu XS, Pettit C, Dillhoff M, Manilchuk A, Schmidt C, Wuthrick E, Chen W, Williams TM (2017). Prognostic value of microRNA expression levels in pancreatic adenocarcinoma: a review of the literature. Oncotarget..

[21] Deng Z, Rong Y, Teng Y, Zhuang X, Samykutty A, Mu J, Zhang L, Cao P, Yan J, Miller D, Zhang HG (2017). Exosomes miR-126a released from MDSC induced by DOX treatment promotes lung metastasis. Oncogene..

[22] Genovese I, Fiorillo A, Ilari A, Masciarelli S, Fazi F, Colotti G (2017). Binding of doxorubicin to Sorcin impairs cell death and increases drug resistance in cancer cells. Cell Death Dis.

[23] Ettinger DS, Wood DE, Aisner DL, Akerley W, Bauman J, Chirieac LR, D'Amico TA, DeCamp MM, Dilling TJ, Dobelbower M, Doebele RC, Govindan R, Gubens MA, Hennon M, Horn L, Komaki R, Lackner RP, Lanuti M, Leal TA, Leisch LJ, Lilenbaum R, Lin J, Loo BW, Martins R, Otterson GA, Reckamp K, Riely GJ, Schild SE, Shapiro TA, Stevenson J, Swanson SJ, Tauer K, Yang SC, Gregory K, Hughes M (2017). Non-small cell lung cancer, Version 5.2017, NCCN Clinical Practice Guidelines in Oncology. J Natl Compr Cancer Netw.

[24] Daniel C, Mato AR (2017). BCL-2 as a therapeutic target in chronic lymphocytic leukemia. Clin Adv Hematol Oncol.

[25] Li Z, Ying X, Chen H, Ye P, Shen Y, Pan W, Zhang L (2014). MicroRNA-194 inhibits the epithelial-mesenchymal transition in gastric cancer cells by targeting FoxM1. Dig Dis Sci.

[26] Miao J, Wang W, Wu S, Zang X, Li Y, Wang J, Zhan R, Gao M, Hu M, Li J, Chen S (2018). miR-194 suppresses proliferation and migration and promotes apoptosis of osteosarcoma cells by targeting CDH2. Cell Physiol Biochem.

[27] Wang ZH, Ren LL, Zheng P, Zheng HM, Yu YN, Wang JL (2014). miR-194 as a predictor for adenoma recurrence in patients with advanced colorectal adenoma after polypectomy. Cancer Prev Res.

[28] Wu J, Zhang L, Wu S, Yi X, Liu Z (2020). miR-194-5p inhibits SLC40A1 expression to induce cisplatin resistance in ovarian cancer. Pathol Res Pract.

[29] Nakamura K, Sawada K, Miyamoto M, Kinose Y, Yoshimura A, Ishida K, Kobayashi M, Shimizu A, Nakatsuka E, Hashimoto K, Mabuchi S, Kimura T (2019). Downregulation of miR-194-5p induces paclitaxel resistance in ovarian cancer cells by altering MDM2 expression. Oncotarget..

